# Non-invasive Predictors of Esophageal Varices

**DOI:** 10.4103/1319-3767.74470

**Published:** 2011

**Authors:** Jijo V. Cherian, Nandan Deepak, Rajesh Prabhu Ponnusamy, Aravindh Somasundaram, V. Jayanthi

**Affiliations:** Department of Gastroenterology, Stanley Medical College Hospital, Chennai, India; 1 Department of Medicine, Stanley Medical College Hospital, Chennai, India

**Keywords:** Esophageal varices, non-invasive predictors, portal hypertension

## Abstract

**Background/Aim:**

Current guidelines recommend screening cirrhotic patients with an endoscopy to detect esophageal varices and to institute prophylactic measures in patients with large esophageal varices. In this study, we aimed at identifying non-endoscopic parameters that could predict the presence and grades of esophageal varices.

**Patients and Methods:**

In a prospective study, 229 newly diagnosed patients with liver cirrhosis, without a history of variceal bleeding, were included. Demographic, clinical, biochemical and ultrasonographic parameters were recorded. Esophageal varices were classified as small and large, at endoscopy. Univariate analysis and multivariate logistic regression analysis were done to identify independent predictors for the presence and grades of varices.

**Results:**

Of the 229 patients (141 males; median age 42 years; range 17-73 years) with liver cirrhosis, 97 (42.3%) had small and 81 (35.4%) had large varices. On multivariate analysis, low platelet count (Odd’s Ratio [OR], 4.3; 95% confidence interval [CI], 1.2-14.9), Child Pugh class B/C (OR, 3.3; 95% CI, 1.8-6.3), spleen diameter (OR, 4.3; 95% CI, 1.6-11.9) and portal vein diameter (OR, 2.4; 95% CI, 1.1-5.3) were independent predictors for the presence of varices. Likewise, for the presence of large esophageal varices, low platelet count (OR, 2.7; 95% CI, 1.4-5.2), Child Pugh class B/C (OR, 3.8; 95% CI, 2.3-6.5) and spleen diameter (OR, 3.1; 95% CI, 1.6-6.0) were the independent risk factors.

**Conclusion:**

The presence and higher grades of varices can be predicted by a low platelet count, Child-Pugh class B/C and spleen diameter. These may be considered as non-endoscopic predictors for the diagnosis and management of large grade varices.

Esophageal varices develop as a consequence of portal hypertension in patients with chronic liver disease and are present in approximately 50% of patients with cirrhosis of the liver. The grade of esophageal varices often correlates with the severity of liver disease. While approximately 85% of individuals with Child-Pugh C cirrhosis have varices, they are present in only 45% those with Child-Pugh A cirrhosis.[1] The rate of development of new varices and increase in grades of varices is 8% per year; the former is largely predicted by a hepatic venous pressure gradient (HVPG) exceeding 10 mm Hg[2,3] and the latter by the presence of decompensated cirrhosis, alcohol etiology and red wale signs.[3]

Large size varices, the presence of red color signs, severe liver disease and portal pressure greater than 12 mm Hg[4,5] predict greater risk of bleeding. Mortality rate of an episode of esophageal varices bleeding is approximately 20% at six weeks.[6,7]

Predicting the grade of varices by non-invasive methods at the time of registration is likely to predict the need for prophylactic β blockers or endoscopic variceal ligation in patients with cirrhosis and portal hypertension. Therefore, the present study has been undertaken to determine the appropriateness of the various clinical, biochemical and imaging parameters in predicting the existence and also the grade of esophageal varices in cirrhosis of the liver.

## PATIENTS AND METHODS

Consecutive newly diagnosed patients with cirrhosis of the liver, presenting to the liver clinic of our centre, between July 2004 and December 2007, were included in this prospective study. Individuals presenting with variceal bleed, those with a past history of bleed and who had undergone sclerosis or band ligation of esophageal varices, portal vein thrombosis, hepatoma, or on current or past treatment with beta-adrenergic receptor blockers were excluded from the study. All the patients underwent detailed clinical evaluation, appropriate investigations, imaging studies (ultrasound with Doppler) and endoscopy at our centre. Diagnosis of cirrhosis was based on clinical, biochemical and ultrasonographic findings.[8]

History included details and duration of alcoholism, jaundice, ascites, oliguria, pedal edema and gastrointestinal bleed. Presence or absence of jaundice, ascites, splenomegaly and hepatic encephalopathy was noted. Hemoglobin, platelet count, prothrombin time, blood urea, serum creatinine, blood glucose, liver function tests including serum bilirubin, albumin/globulin ratio and transaminases were estimated. Modified Child-Turcotte-Pugh (CTP) class was calculated for each patient. Special investigations included HBsAg, anti HCV antibody assay, slit lamp examination, serum Ceruloplasmin, 24 h urine copper, iron studies, antinuclear antibody, anti smooth muscle antibody and antimitichondrial antibody assays. Patients with an alcohol etiology were included in the study after at least six months of abstinence.

At ultrasonogram and Doppler study (VJ), the portal vein and spleen diameter along with echo texture of the liver and direction of blood flow were noted. The portal vein diameter and platelet count / spleen diameter ratio were determined. Coefficient of variation for repeated measurements of these parameters was less than 2%. All endoscopies were performed in a single endoscopy unit using a video-endoscope. At endoscopy, the esophageal varices were graded as large (Grade III-IV) or small (Grade I-II), based on Paquet’s grading system.[9] The endoscopist and the sonologist were blinded to the clinical and laboratory parameters.

### Statistical analysis

Univariate analyses were done using the Mann-Whitney U-test for continuous variables and the χ^2^ test for categorical variables. A *P* value of less than 0.05 was considered as significant. All variables that were found to be of significance on univariate analysis were included as candidate variables in a forward-conditional step-wise logistic regression analysis to identify independent predictors for the presence of esophageal varices and their size.

**Figure 1:**
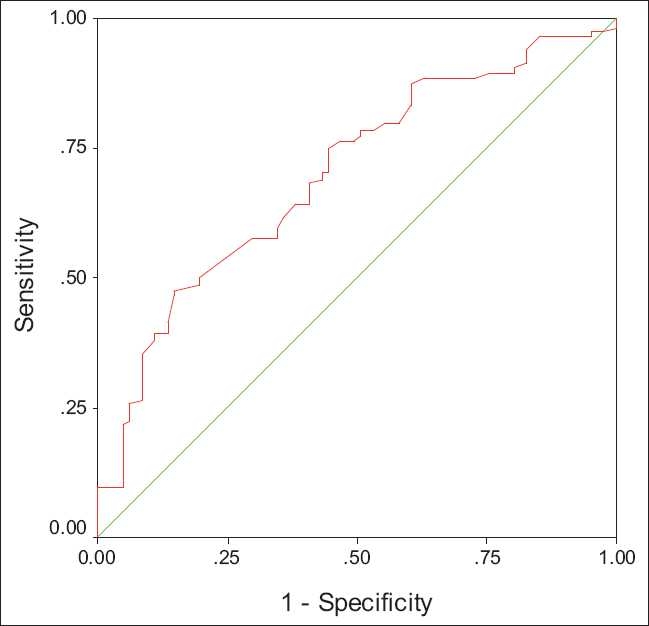
ROC curve for platelet count in predicting large esophageal varices (AUROC: 0.70)

**Figure 2:**
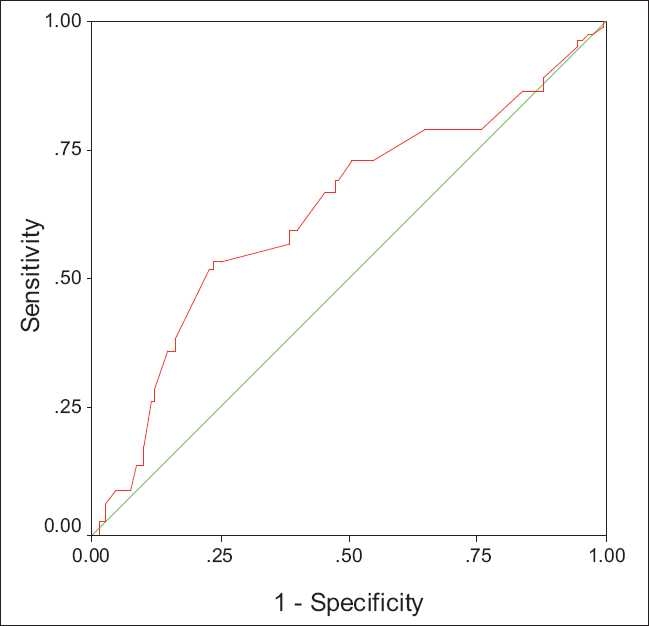
ROC curve for spleen bipolar diameter in predicting large esophageal varices (AUROC: 0.63)

Receiver operating characteristic curves (ROC curves) were applied to find the best sensitivity and specificity cut off values of the continuous variables for the presence or absence of esophageal varices and for the presence of large esophageal varices [Figures 1 and 2].

## RESULTS

Two hundred and twenty nine eligible patients (141 males; median age 42 years; range 17-73 years) with cirrhosis of the liver were included in the study. The etiology included alcohol (97 patients, 42.4%) followed by hepatitis B virus (35 patients; 15.3%) and hepatitis C virus (23 patients; 10%) infection. Other/mixed causes were non alcoholic fatty liver disease: 10, alcohol and HCV: 4, alcohol and HBV: 3, Budd-Chiari syndrome: 4, Wilson’s disease: 3, autoimmune hepatitis: 3, HBV and HCV: 1, secondary biliary cirrhosis: 1 and the cause remained unknown in 45 patients (19.7%).

There were 42 patients in CTP class A and 187 in class B or C (127 in class B; 60 in class C). Overall, 51 patients had no esophageal varices (22.3%), 97 (42.3%) had small varices (Gr I-II) and 81 (35.4%) had large varices (Gr III-IV).

### a. Risk factors for the presence of any grades of varices

On univariate analysis, CTP class (B/C), platelet count, prothrombin time, spleen diameter, portal vein diameter and platelet count /spleen diameter ratio were significantly associated with the presence of esophageal varices [Table 1]. On multivariate analysis, the presence of esophageal varices was significantly associated with CTP class B/C (OR 3.3; 95% CI, 1.8 – 6.3), platelet count < 100,000/ μl (OR 4.3; 95% CI, 1.2 – 14.2), spleen diameter > 150 mm (OR 4.3; 95% CI, 1.6 - 11.8) and portal vein diameter > 13 mm (OR 2.4; 95% CI, 1.1 – 5.3) [Table 2].

**Table 1:** Relationship of various parameters with the presence or absence of esophageal varices on univariate analysis

Variable	Varices absent (*n*=51) Median (range)	Varices present (*n*=178) Median (range)	*P* value
Male/ female	38/13	104/75	NS*
Median age (range)	42 (17–73)	42 (17–70)	NS
Ascites	27	105	NS
Splenomegaly	25	106	NS
Hepatic Encephalopathy	10	33	NS
Child Pugh class(A vs B/C)			0.001
Class A	17	25	
Class B	32	95	
Class C	2	58	
Hemoglobin (g/dl)	9.1 (4.6–17)	9.8 (4.8–17.2)	NS
S bilirubin (mg/dl)	1 (0.4–8.8)	1.2 (0.6–14.6)	NS
S albumin (g/dl)	3 (1.6–5)	2.8 (1.7–5)	NS
Prothrombin time (seconds prolonged)	5 (0–10)	7 (0–28)	0.01
Platelet count (per μl)	152000 (15000– 402000)	90000 (28000– 276000)	0.001
Spleen bipolar diameter (mm)	150 (70–240)	170 (85–300)	0.001
Portal vein diameter (mm)	11 (8–23)	14 (9–23)	0.005
Platelet count/spleen diameter ratio	962.03 (115.38– 4466.67)	555.56 (96.55– 1916.67)	0.001

* NS: Not significant

**Table 2:** Multivariate logistic regression analysis for the presence or absence of varices using Enter method

Parameter	*P* value	Odds ratio	95% confidence interval	
			Lower	Upper
CTP class (A vs B/C)	0.000	3.311	1.753	6.255
Platelet count (< 1,00,000/μl)	0.021	4.292	1.242	14.925
Spleen bipolar diameter (>150 mm)	0.004	4.334	1.582	11.874
Portal vein diameter (>13 mm)	0.026	2.421	1.111	5.277

On multivariate analysis, the platelet count-spleen diameter ratio was not predictive for the presence of varices; however, a cut-off value of ≤ 666 had a sensitivity of 66.3%, specificity of 80.4%, positive predictive value of 92.2% and a negative predictive value of 40.6% for the presence of esophageal varices.

### b. Risk factors for the presence of large varices

The predictors of large varices on univariate analysis were CTP class B/C, platelet count, spleen bipolar diameter, portal vein diameter and platelet count /spleen diameter ratio [Table 3]. On multivariate analysis [Table 4], the significant factors included CTP class B or C (OR 3.8; 95% CI, 2.3 – 6.5), platelet count < 90,000/ *μ*l (OR 2.7; 95% CI, 1.4 – 5.2) and spleen diameter > 160 mm (OR 3.1; 95% CI, 1.6 – 6).

**Table 3:** Relationship of various parameters with the presence or absence of large esophageal varices on univariate analysis

Variable	Small (Grade I-II) or no varices (n=148)	Large varices (Grade III-IV) (n=81)	*P* value
Male/ female	88/60	53/28	NS
Median age (range)	41 (17–73)	43 (17–67)	
Ascites	83	49	NS
Splenomegaly	77	53	NS
Hepatic encephalopathy	19	24	NS
CTP class (A vs B/C)			0.001
Class A	38	4	
Class B	89	38	
Class C	21	39	
Hemoglobin (g/dl)	9.2 (4.9–17.2)	9.8 (4.8–14.2)	NS
S albumin (g/dl)	3.0 (1.6–5.0)	2.7 (1.8–3.9)	NS
S bilirubin (mg/dl)	1.1 (0.4–14.6)	2 (0.8–10)	NS
Prothrombin time (seconds prolonged)	6 (0–20)	8 (0–28)	NS
Platelet count (per μl)	104500 (15000– 402000)	78000 (28000– 219000)	0.001
Spleen bipolar diameter (mm)	155 (70–300)	180 (85–290)	0.001
Portal vein diameter (mm)	13 (9–23)	14 (10–23)	0.005
Platelet count/ spleen diameter ratio	699.33 (115.38– 4466.67)	462.50 (96.55– 1078.81)	0.001

**Table 4:** Multivariate logistic regression analysis for the presence of large varices using Enter method

Parameter	*P* value	Odds ratio	95% confidence interval	
			Lower	Upper
CTP class (A vs B/C)	0.000	3.842	2.277	6.483
Platelet count (< 90,000/μl)	0.003	2.695	1.397	5.181
Spleen bipolar diameter (>160 mm)	0.001	3.108	1.612	5.992

### c. Correlation of predictors of large varices with endoscopy grading

Using the significant factors on multivariate analysis, which predicted large varices, CTP class was the most sensitive in picking up large grade varices at endoscopy (sensitivity: 95%; specificity 26%). When all the three parameters i.e CTP score, platelet count and spleen diameter were considered together, the sensitivity was low but specificity was high (sensitivity: 33% and specificity: 92%). CTP class B/C had the lowest miss rate for large varices (9.5%), and the gastroscopies saved was 16.6%. Other parameters had an unacceptable level of miss rates for large esophageal varices (>20%) [Table 5].

**Table 5:** Sensitivity, specifi ty, positive and negative predictive values and the EGD’s saved (%) for the signifi cant parameters

Parameter	Sensitivity (%)	Specifi city (%)	PPV (%)	NPV (%)	EGD’s saved (%)
CTP class B/C	95	25.7	41.2	90.5	16.6
Spleen diameter ≥ 160 mm	66.7	54.7	44.6	75	41.5
Platelet count ≤ 90,000 /mm^3^	59.3	64.2	47.5	74.2	35.4
Presence of all of the above three parameters	33	91.9	69.2	71.6	59.4

PPV: Positive predictive Value; NPV: Negative predictive value,

EGD: Esophagogastroduodenoscopy

## DISCUSSION

Several studies in the past have shown independent parameters like splenomegaly,[10,11,17,18,22] ascites,[12,18] spider naevi,[13] Child’s grade,[15] platelet count,[11–18,20,22] prothrombin time/activity,[13,16] portal vein diameter,[16] platelet count/ spleen diameter ratio,[19,21] serum albumin,[20] and serum bilirubin[20] as significant predictors for the presence of esophageal varices.

The present study further corroborates the results of earlier studies. Giannini *et al*,[19] proposed the platelet count-spleen diameter ratio of ≤ 909, as an accurate non-invasive marker for the presence of esophageal varices. This was further validated in a multicenter trial.[21] The study population comprised predominantly of patients with hepatitis C related cirrhosis. A similar study by Agha *et al*,[23] from Pakistan, made identical observations in the same subset of patients. Sen *et al*,[24] found the platelet count-spleen diameter ratio of ≤ 650 as a sensitive non-invasive marker [Area under curve (AUC) of 0.81] in HCV related cirrhosis.

In the present study, on univariate analysis, a platelet count-spleen diameter ratio of ≤ 666 was significantly associated with the presence of esophageal varices in a predominant alcohol related cirrhosis subset. This ratio was insignificant on multivariate analysis. Sen *et al*,[24] made similar observations (AUC of 0.75 in alcohol related cirrhosis versus AUC of 0.81 for HCV related cirrhosis).

Non endoscopic assessment for presence and grades of varices from India are few. Amarapurkar *et al*,[10] report that splenomegaly alone was a significant predictor for the development of large esophageal varices. Sharma *et al*,[22] in a prospective study, observed that splenomegaly and platelet count were the independent predictors for the presence of large varices. They could derive a predictor function based on this observation, which had an AUC of 0.76.

**Figure 3:**
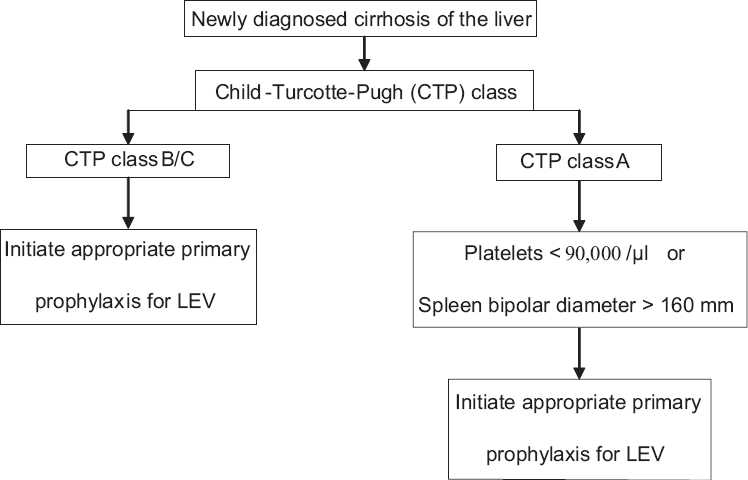
Algorithm for the initiation of primary prophylaxis for large esophageal varices (LEV) based on the present study

From the present study, Child Pugh class B/C, low platelet count and spleen diameter emerged as significant predictors for the presence of large esophageal varices. Of these variables, CTP class B/C missed less than 10% of patients with large varices and saved one endoscopy procedure for every six procedures performed. Four out of forty-two patients in CTP class A had large varices. All the four patients had either a platelet count of < 90,000/μl or spleen bipolar diameter > 160 mm. The following algorithm [Figure 3] could be suggested for the initiation of primary prophylaxis for large esophageal varices, based on the non endoscopic parameters, from the present study.

We believe that these predictors may be of help to the physicians practicing in rural areas where endoscopy facilities are not readily available, in helping them to initiate appropriate primary pharmacological prophylaxis in these patients. In an urban setting where the endoscopy workload is high, a non invasive predictor, as in this study, can help one to initiate drug therapy while waiting for the endoscopy procedure.
